# Selective Estrogen Receptor Down-Regulator and Selective Estrogen Receptor Modulators Differentially Regulate Lactotroph Proliferation

**DOI:** 10.1371/journal.pone.0010060

**Published:** 2010-04-19

**Authors:** Sanjay Kansra, Shenglin Chen, Madhavi Latha Yadav Bangaru, Leighton Sneade, Joseph A. Dunckley, Nira Ben-Jonathan

**Affiliations:** 1 Department of Cancer and Cell Biology, University of Cincinnati College of Medicine, Cincinnati, Ohio, United States of America; 2 Department of Endocrinology, Metabolism and Clinical Nutrition, Medical College of Wisconsin, Milwaukee, Wisconsin, United States of America; 3 Department of Pharmacology, Medical College of Wisconsin, Milwaukee, Wisconsin, United States of America; Ecole Normale Supérieure de Lyon, France

## Abstract

**Background:**

We recently reported that estrogen receptor α (ERα), even in absence of estrogen (E2), plays a critical role in lactotroph homeostasis. The anti-estrogen ICI 182780 (ICI), but not tamoxifen or raloxifene, rapidly promoted the degradation of ERα, and inhibited cell proliferation. However, all three ER antagonists suppressed PRL release, suggesting that receptor occupation is sufficient to inhibit *prl* gene expression whereas receptor degradation is required to suppress lactotroph proliferation. In this study our objective was to determine whether ERα degradation *versus* occupation, differentially modulates the biological outcome of anti-estrogens.

**Principal Findings:**

Using the rat lactotroph cell line, GH3 cells, we report that ICI induced proteosome mediated degradation of ERα. In contrast, an ERα specific antagonist, MPP, that does not promote degradation of ERα, did not inhibit cell proliferation. Further, ICI, but not MPP, abolished anchorage independent growth of GH3 cells. Yet, both ICI and MPP were equally effective in suppressing *prl* expression and release, as well as ERE-mediated transcriptional activity.

**Conclusion:**

Taken together, our results demonstrate that in lactotrophs, ERα degradation results in decreased cell proliferation, whereas ERα occupation by an antagonist that does not promote degradation of ERα is sufficient to inhibit *prl* expression.

## Introduction

Drugs that block estrogen receptor (ER) activation/function are categorized as anti-estrogens. Within this class of pharmacological agents are the selective ER modulators (SERMs) exemplified by tamoxifene (Tam) and raloxifene (Ral), selective ER downregulators, (SERDs) exemplified by ICI 182780 (ICI), and aromatase inhibitors, which inhibit the conversion of androgens to estrogens and block ER activation. Since ICI is deprived of any estrogenic activity [1] it is considered a “pure” anti-estrogen. Several mechanisms have been proposed to characterize ICI-mediated ER antagonism. These include competition with ligand binding to the ER, inhibition of transactivation domains (AF-1 and AF-2), prevention of ER dimerization and nuclear localization [2], [3], and downregulation of ER [4]. Furthermore, both ERα- and ERβ-mediated transcription is inhibited by ICI, indicating that both receptor subtypes are targets of ICI [5], [6].

A response to estrogen is governed by ER availability. Since the initial observation of a reduction of ER expression following exposure to E2 [7], it is now well accepted that the ER protein is rapidly turned over by both agonist and antagonist and its half life is reduced from 24 hr to 3–5 hr in the presence of estrogen [8], [9]. This degradation of the ER is attributed to the processing of the ER *via* the ubiquitin-proteasomal pathway. Blockade of estrogen-induced ER degradation reduces its transcriptional activity, suggesting that receptor processing is required for ER function [10]. The ER is not unique in this respect as other members of the nuclear receptor super-family also require degradation by ubiquitin-proteasome for activation [11], [12]. To further support this hypothesis, several proteins (UBC9, RSP5/RPF1, SUG1/TRIP1 and E6-AP) that interact with the nuclear receptors belong to the ubiquitin-proteasome pathway [13]–[16]. This suggests that agonist-mediated ER degradation, although required for transcriptional activation, could also be a mechanism by which the cell regulates its responses to estrogens. The pure anti-estrogen ICI also rapidly degrades the ER via the ubiquitin-proteasome pathway and thus abolishes the estrogen responsiveness of target cells [4], [10]. Interestingly, tamoxifen stabilizes the ER by inhibiting receptor degradation [17]. Taken together, these observations highlight the importance of regulating the ubiquitin-proteasome pathway, by both estrogen and anti-estrogens, as a critical process for governing ER availability, and ultimately its biological outcome.

Lactotrophs are a well established estrogen-responsive cell. Both genomic and non-genomic effects of E2 have been reported in lactotrophs. Previous reports showed that ICI suppressed cell proliferation and affected ER expression in GH3 and PR1 cells [18], [19]. We conducted a detailed comparison of the effects of ICI, tamoxifen and raloxifene, in the absence of exogenous E2, on lactotroph proliferation and PRL production/release [20]. We found that ICI, but not tamoxifen or raloxifene, at low doses inhibited lactotroph proliferation in an ERα-dependent manner. The maintenance of basal intracellular PRL levels and PRL release were dependent on functional ERα. A striking observation of this study was the very rapid (within 1 hr) reduction in ERα levels, but a significantly delayed reduction in ERβ levels, in response to ICI. The anti-estrogens, tamoxifen and raloxifene, that were incapable of inhibiting lactotroph proliferation, did not downregulate ERα/β [20].

Our objectives in the present study were: first, to determine whether ICI-mediated ERα degradation or antagonism was responsible for inhibiting lactotroph proliferation and PRL expression. Second, to determine whether the disruption of ICI-mediated ERα degradation reverses the growth inhibition in lactotrophs. We report that in GH3 cells ERα degradation sets in motion a signal cascade that culminates in the inhibition of cell proliferation, while occupation of ERα by an antagonist is sufficient to inhibit *prl* expression and release.

## Results

### Differential effects of ERα antagonists on lactotroph proliferation

We have recently reported that anti-estrogens had differential effects on lactotroph proliferation[20]. While tamoxifen and raloxifene had no growth suppressive effects on lactotroph proliferation, the pure ER antagonist, ICI, had a potent growth suppressive effect. We also found that the ability of ICI to suppress lactotroph proliferation was mediated through ERα [20]. In this study, we first questioned whether an ERα specific antagonist mimics the effects of ICI on ERα expression and lactotroph proliferation.

**Figure 1:**
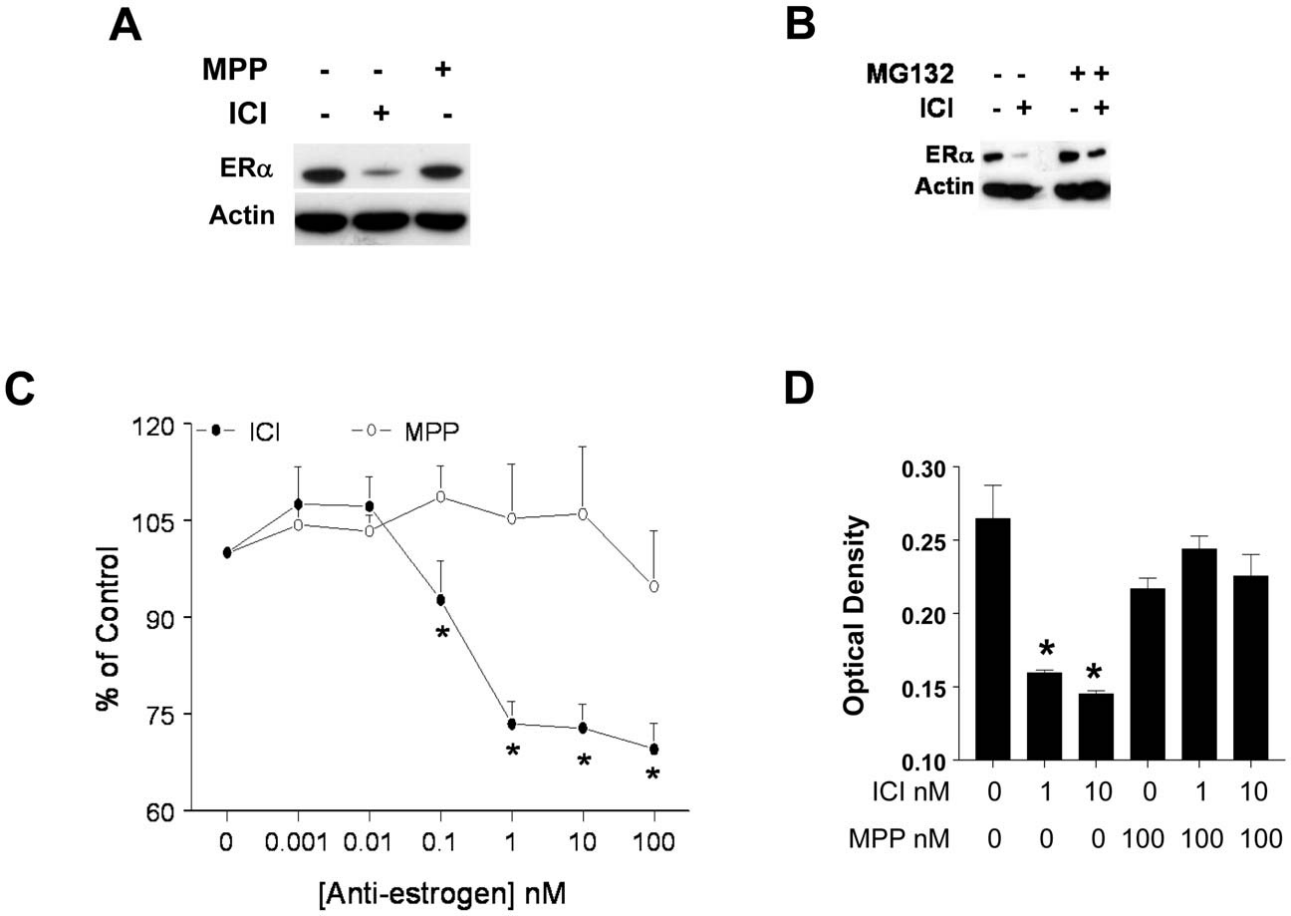
Differential effects of ERα antagonist on cell proliferation and ERα degradation. *A]* GH3 cells were treated with either vehicle, ICI (10 nM) or MPP (100 nM) for 5 days. Cell lysates were subjected to western blotting with an anti-ERα Ab (*top panel*). Equal loading was verified by using the anti-actin Ab (*lower panel*). Results shown are from a single experiment and it is a representative of 3 independent experiments yielding similar results. *B]* GH3 cells were treated either with 10 nM ICI for 1 hr, or pretreated with a broad-spectrum proteosome inhibitor MG132 (10 µM) for 1 hr, followed by ICI for 1 hr. Western blotting using anti-ERα Ab *(Upper Panel)*, or anti-actin Ab *(Lower Panel)*, was performed. Data is from a single experiment and is representative of 2 independent experiments yielding similar results. *C]* GH3 cells were treated with either vehicle or ICI (0.001, 0.01, 0.1, 1, 10 and 100 nM) or MPP (0.001, 0.01, 0.1, 1, 10 and 100 nM) for 5 days, and cell proliferation was determined as described in the materials and methods. Data is expressed as % of Control and it is the mean ± SEM of 3 separate experiments. * indicates significant differences from control (*p<0.05*). *D]* GH3 cells were treated with either vehicle, ICI (1 and 10 nM) or pretreated with MPP (100 nM for 1 hr) followed by treatment with ICI (1 and 10 nM) for 5 days. Cell proliferation was determined, and data is expressed as optical density, and it is the mean ± SEM of 4–6 determinations from a single experiment, which is a representative of 3 separate experiments with similar results. * designates significant differences from control (*p<0.05*).

GH3 cells were treated with either vehicle, ICI (10 nM) or the ERα specific antagonist MPP (100 nM) [21], [22], for 5 days, and equal amounts of cell lysates were subjected to western blotting with an anti-ERα specific Ab. Consistent with our previous report [20], ICI caused a robust degradation of ERα. Unlike ICI, MPP had no significant effect on ERα levels after 5 days (Fig. 1A).

Next, we questioned whether the rapid downregulation of ERα levels in GH3 cells by ICI is mediated through the ubiquitin-proteasome system. Untreated GH3 cells, or cells pretreated with the broad spectrum proteasome inhibitor MG132 (10 µM, 1 hr), were incubated with either vehicle or 10 nM ICI for 1 hr, and equal amount of cell lysate was subjected to western blotting. Fig. 1B shows that in the absence of MG132, ICI induced a potent ERα degradation, and this was significantly reversed in the presence of MG132. Together, these results suggest that ICI induced degradation of ERα could be the underlying mechanism for growth suppression.

We next compared the ability of ICI and MPP to suppress GH3 cell proliferation. GH3 cells were treated with either vehicle or the indicated concentrations of ICI or MPP, and cell proliferation was determined after 5 days. Fig. 1C clearly demonstrates that consistent with our previous report, ICI inhibits GH3 cell proliferation with a maximal inhibitory effect seen at concentrations as low as 1 nM. Conversely, MPP at all tested concentrations had no significant effect on GH3 cell proliferation.

Since both MPP and ICI competitively inhibit E2 binding to ER, we questioned whether pretreatment of GH3 cells with MPP would prevent ICI binding to ERα, thereby blocking its effects. To address this, we pretreated GH3 cells with excess MPP (100 nM for 1 hr) and then tested the ability of ICI (1 and 10 nM) to inhibit cell proliferation. Our results demonstrate (Fig. 1D) that, pretreatment of GH3 cells with MPP, reversed the inhibitory effect of ICI on cell proliferation.

### ICI 182780 but not MPP inhibits anchorage independent growth of GH3 cells

**Figure 2:**
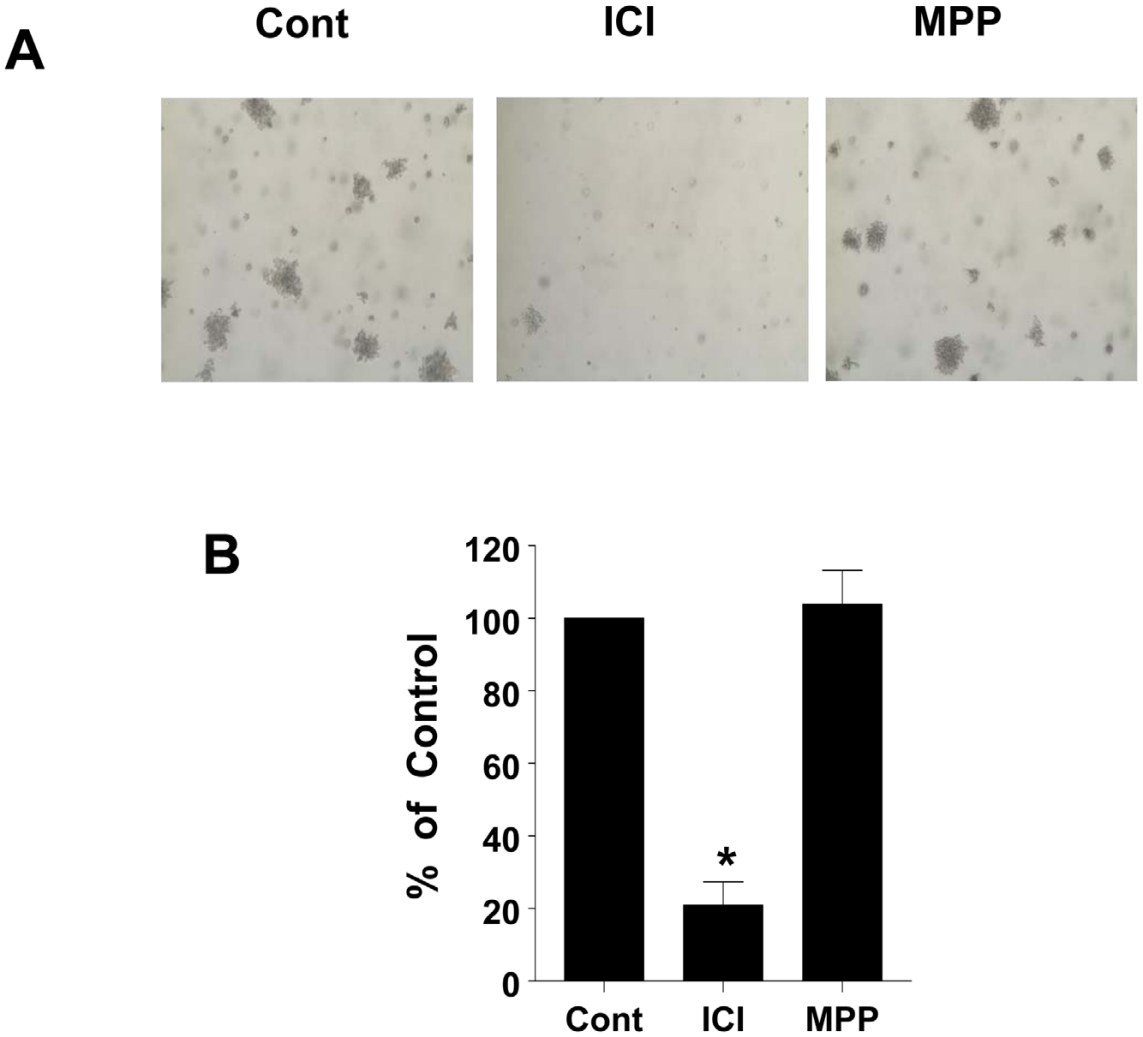
ICI 182780 but not MPP inhibits anchorage independent growth of GH3 cells. GH3 cells were cultured on soft agar in complete medium containing ICI (10 nM) or (MPP 10 nM) as described in the materials and methods. *A]* Bright field microscopy shows GH3 cell colonies in soft agar. *B]* Quantitative analysis of GH3 cell colonies in soft agar following anti-estrogen treatment. Colonies were counted in at least 3 independent fields. Data is calculated as % of control and it is the mean ± SEM of 4 separate experiments. * indicates significant differences from control (*p<0.05*).

We next compared the effects of the ERα antagonists on the anchorage independent growth of GH3 cells. Our results demonstrate that GH3 cells have a robust capability to form colonies in soft agar, and this was significantly decreased by ICI (Control 100% *vs* ICI 20.9%) whereas MPP had no effect on anchorage independent growth of GH3 cells (Fig. 2A and B). The effects of the anti-estrogens in the anchorage independent growth assays were similar to those observed in clonogenic assays with GH3 cells (data not shown).

### Both ICI 182780 and MPP are effective at inhibiting PRL expression as well as suppressing ERE activity

**Figure 3:**
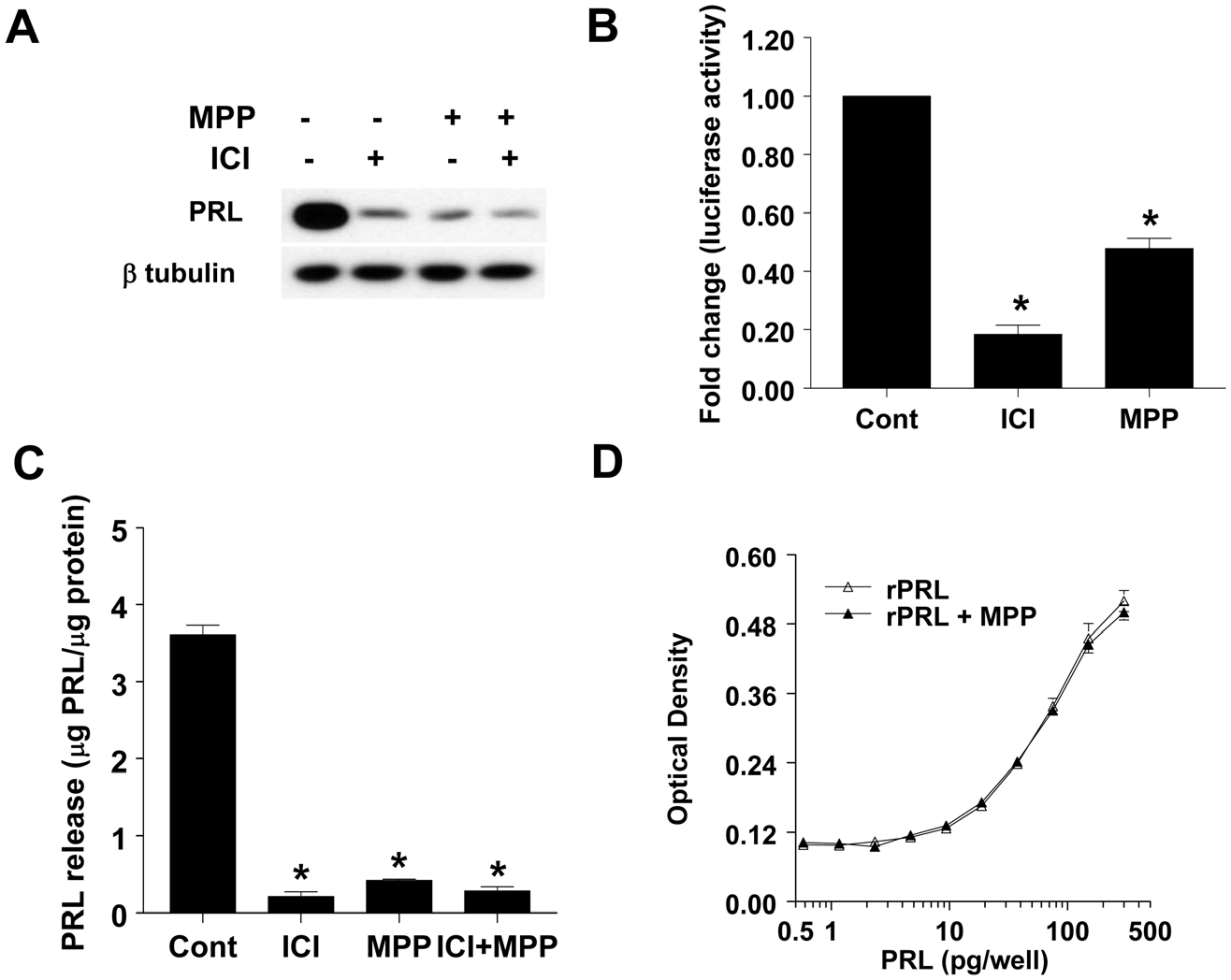
MPP and ICI 182780 inhibit PRL production and release. *A]* GH3 cells were treated with either vehicle, or ICI (10 nM), or MPP (100 nM) or ICI + MPP for 5 days and cell lysates were subjected to western blotting using anti-PRL Ab (*top panel*) or anti-β tubulin Ab (*bottom panel*). Data presented is from a single experiment and is representative of 3 separate experiments yielding similar results. *B]* GH3 cells, transiently co-transfected with PRL-Luc and control reporter gene, were treated with either vehicle, or ICI (10 nM), or MPP (10 nM) for 24 hrs, and normalized luciferase activity was determined. Data is calculated as fold change over control (arbitrary value of 1). Each value is the mean ±SEM of 3 separate experiments each performed in triplicates. * indicates significant difference from control, (*p<0.05*). *C]* GH3 cells were treated for 5 days with either vehicle, or ICI (10 nM), or MPP (100 nM) or ICI + MPP and the amount of PRL released into the CM was quantified by the Nb2 bioassay and is expressed as µg PRL/µg protein. Each value is the mean ±SEM of 3 determinations from a single experiment, which is representative of 3 separate experiments. * indicates significant difference from control (p<0.05). *D]* Nb2 cells were incubated with rPRL or rPRL in presence of MPP, and Nb2 cell proliferation at the end of 3 days was determined by the MTT assay. Data is expressed as optical density, and it is the mean ± SEM of 3 determinations from a single experiment, which is a representative of 3 separate experiments with similar results.

We next examined whether occupation of ERα by an antagonist or ERα degradation, are required for inhibiting PRL production and release. We first examined the intracellular levels of PRL in GH3 cells treated with ICI (10 nM), MPP (100 nM) or preincubated with MPP for 1 hr, followed by ICI treatment. Intracellular PRL levels were determined in cell lysates by western blotting with an anti-PRL Ab. Fig. 3A shows that both ICI and MPP markedly decreased intracellular PRL levels. Pretreatment with MPP did not block the ICI-induced decrease in intracellular PRL, and did not have an additive effect. To further confirm the suppressive effects of anti-estrogens on intracellular PRL levels, we used a reporter assay to examine the effects of ICI and MPP on *prl* expression. As evident in Fig. 3B, both ICI and MPP suppressed PRL/Luc activity.

We next examined the effects of the anti-estrogens on PRL release from GH3 cells. GH3 cells were treated with ICI (10 nM), MPP (100 nM), or pretreated with MPP (for 1 hr) followed by ICI. After 48 hrs, PRL released into the CM was determined by the sensitive Nb2 bioassay. (Fig. 3C) clearly shows that both ICI and MPP independently inhibit PRL release from GH3 cells. Pretreatment with MPP did not lead to blockade or augmentation of the inhibitory effects of ICI on PRL release. We have previously shown that the presence of ICI in the CM, at the dilutions used in the Nb2 assay, does not directly affect the proliferation of Nb2 cells [20]. To verify that the observed MPP-induced decrease in PRL released (Fig. 3C) was not due to its direct effect on Nb2 cells, we examined the responsiveness of Nb2 cells to rat PRL in the presence and absence of MPP. Our results (Fig. 3D) show MPP had no significant effect on Nb2 cell proliferation in response to PRL.

**Figure 4:**
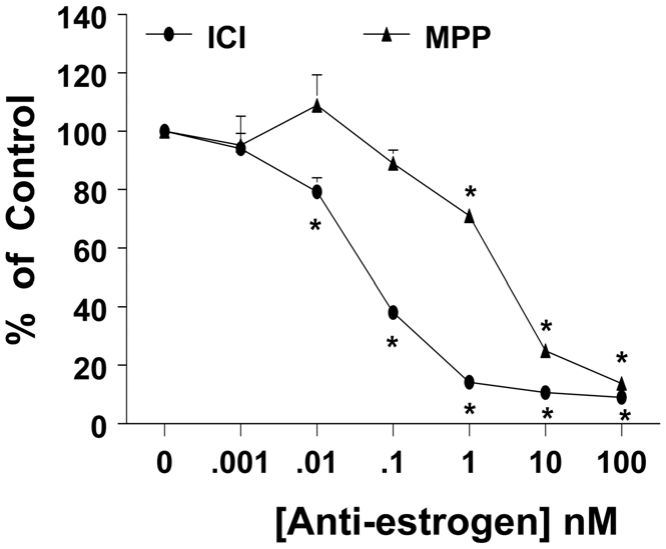
MPP and ICI 182780 inhibit ERE transcriptional activity. GH3 cells, transiently co-transfected with an ERE reporter gene and a control reporter gene, were treated with the indicated concentrations of ICI or MPP for 24 hrs, and normalized luciferase activity was determined as described in methods. Data was calculated as % of vehicle control (100%). Each value is the mean ±SEM of 4 separate experiments each performed in triplicates. * indicates significant difference from control (*p<0.05*).

Since both ICI and MPP suppressed *prl* expression, we next questioned whether either compound suppresses ERE-mediated transcriptional activity. For that, GH3 cells were transfected with ERE-Luc reporter plasmid, and the ability of ICI and MPP to suppress ERE transcriptional activity were evaluated. Fig. 4 demonstrates that Both ICI and MPP suppress ERE transcriptional activity in a dose dependent manner, with a significant (p<0.05) suppression seen with 0.1 nM of ICI and 1 nM of MPP. At the 10 nM concentration, both ICI (89.4% inhibition) and MPP (75% inhibition) robustly suppressed ERE activity.

## Discussion

Work from our laboratory as well as others have established a role for ER in mediating lactotroph proliferation and PRL expression, both in the presence and absence of E2. We have previously shown that, even in the absence of E2, the “pure” anti-estrogen ICI caused a rapid and robust degradation of ERα, leading to inhibition of cell proliferation. We also demonstrated that although Tam and Ral failed to significantly inhibit lactotroph proliferation, like ICI, they are potent inhibitors of PRL release from GH3 cells [20]. These studies lead us to hypothesize that differential biological outcomes can be expected, depending upon the ability of the anti-estrogen to degrade ERα.

Our results show that ICI induces proteasome-mediated degradation of ERα in GH3 cells which was reversed by the broad spectrum proteasome inhibitor MG132 (Fig. 1). The ability of ICI to degrade ER *via* the proteasome is consistent with previous reports in a variety of cell types [2], [4]. We next questioned whether preventing ICI-induced ERα degradation limits its growth inhibitory effect on GH3 cells. A simple approach would have been to block the ICI-induced ERα degradation with MG132 (Fig. 1), and then assess its effect on cell proliferation. However, we (data not shown) and others have shown that prolonged use of proteasome inhibitors results in apoptosis in lactotrophs [23]. We further explored this issue by comparing the effects of MPP, a highly specific ERα antagonist that does not degrade ERα [21],[22]. Unlike ICI, which has a rapid and robust effect on ERα degradation, MPP had no effect on ERα degradation. Compared to ICI, MPP by itself was ineffective at inhibiting GH3 cell proliferation. Supporting the above conclusion were the results of the anchorage-independent growth assay as well as clonogenic property of GH3 cells. While ICI completely blocked colony formation, MPP had no significant effect. In addition, preincubation of GH3 cells with 10-fold excess MPP, blocks ICI-induced inhibition of cell proliferation (Fig. 1D). The inability of MPP to suppress cell proliferation as well as anchorage independent growth of GH3 cells was not due to its ineffectiveness, since both ICI and MPP were effective at blocking ERE activity in reporter gene assays (Fig. 4). Further, this differential effect of ICI and MPP were observed in two other rat lactotroph cell lines (data not shown). Taken together, we conclude that in the absence of E2 occupation of ERα by an antagonist is not sufficient to inhibit lactotroph proliferation. Instead, ERα degradation must follow upon antagonist occupation of the receptor.

While both MPP and ICI are competitive inhibitors of ligand binding to ERα, it could be hypothesized that their differential effects on cell proliferation are due to their interaction with different pools of ERα. Although both our work (unpublished observations) as well as work from other laboratories have demonstrated the presence of nuclear as well as extra-nuclear ERα in GH3 cells, it is unlikely that ICI and MPP target different pools of ERα. This is based on our observations that excess MPP effectively blocks the ICI induced inhibition of cell proliferation. An alternate mechanism which could explain the differential growth suppressive effects of ICI and MPP, is an involvement of non-genomic/non-classical signaling mechanisms activated by anti-estrogen-occupied ER. Indeed, ICI-mediated Erk1/2 activation appears to be critical for growth modulation in immature cerebellar neurons [24]. Our preliminary results demonstrate that ICI induced growth suppression of pituitary lactotrophs is accompanied with decrease in cyclin D3 expression and phosphorylation of Rb (unpublished observations). These results are consistent with a previous study demonstrating a decrease in cyclin D3 levels in response to ICI treatment in PR1 pituitary lactotrophs [18]. Future studies will examine whether ICI-mediated growth suppression is due to modulation of a non-genomic signaling pathway that leads to decrease cyclin D3 expression and decreased phosphorylation of Rb.

We next explored the role of ERα degradation on PRL production and release. The use of a reporter assay, western blotting for intracellular PRL levels, and Nb2 assay for secreted PRL we found that both ICI and MPP are equally effective at suppressing PRL gene expression (Fig. 3B), decreased intracellular PRL (Fig. 3A), as well as decreased release of PRL from GH3 cells into CM (Fig. 3C). When GH3 cells were preincubated with a 10 -fold excess of MPP, MPP failed to block the decreased expression of *prl*, intracellular levels of PRL as well as the amount of PRL released into the CM. These results indicate that occupation, and not degradation of ERα, is sufficient to inhibit *prl* expression, and subsequently, its production and release from GH3 cells. Since both ICI and MPP were effective at suppressing *prl* expression, we examined whether either compound inhibits ERE-mediated transcriptional activity. At the 10 nM levels, both ICI and MPP inhibited ERE activity by more than 75%. However, at 10 nM levels, MPP did not have any significant effect on cell proliferation or ERα degradation. This suggests that suppression of ERE transactivation is not the underlying mechanism by which ICI inhibits cell proliferation.

Taken together our results indicate that in pituitary lactotrophs, an ERα degrading antagonist such as ICI, but not an ERα occupying antagonist such as MPP, initiates a signal cascade that inhibits cell proliferation. On the other hand, occupation of ERα by either type of antagonist is sufficient for inhibiting *prl* expression and release. Anti-estrogens have been proposed as a class of novel therapeutics for suppressing prolactionomas. Therefore, a complete understanding of their differential effects on the lactotrophs could help in the development of more effective therapeutics.

## Materials and Methods

### Chemicals and reagents

ICI 182780 and the ERα specific antagonist 4,4′,4″-(4-Propyl-[1H] pyrazole-1,3,5-triyl) trisphenol (MPP), were purchased from Tocris (Ellisville, MO). MG 132 was purchased from Calbiochem/EMD Chemicals. Inc. (La Jolla, CA).

### Cell culture

GH3 cells were purchased from ATCC (Manassas, VA) and maintained in DMEM:F12 50∶50 mix (Mediatech, Herndon, VA) containing 10% FBS(Gibco/Invitrogen) and 5 U/ml Penicillin/5 µg/ml Streptomycin (Pen/Strep).

### Assessment of cell proliferation

GH3 cells in log phase were seeded at 20,000–30,000 cells/well in DMEM:F12 (50∶50) phenol red -free medium containing ITS (insulin, transferrin, selenious acid) premix (BD Biosciences, San Jose, CA) and Pen/Strep. Next day, cells were treated as indicated in plating medium. Cell proliferation was quantitated using MTT assay as described [20]. Briefly, 125 µg/well of MTT (3-[4,5-dimethylthiazole-2-yl] -2,5-diphenyltetrazolium bromide (Sigma) was added to the treatment wells, and 2 hrs later, 100 µl of developer solution (50% v/v DMF; 20% w/v SDS; 0.24% v/v glacial acetic acid; 60 mM sodium acetate) was added. Optical density at 570 nm was determined. Data are presented as optical density or as percent of vehicle (DMSO) control. We have recently shown that the MTT assay is in excellent agreement with BrdU incorporation assay in determining cell proliferation or inhibition in GH3 cells [20].

### Luciferase reporter assays

After seeding, GH3 cells were cultured for 24 hrs, followed by transient co-transfection with 0.8 µg of the 2.5 Kb rat PRL pA3 PRL/luc plasmid [25]; (a gift from Dr. A. Gutierrez-Hartman, Denver, CO); or p3X ERE-luc reporter plasmid (gifted from Dr. R. Bigsby, Indianapolis, IN); together with the control pGL4.70 [hRLuc] Renilla plasmid (Promega, Madison, WI), using Lipofectamine 2000 (Invitrogen). Cells were washed and the medium was replaced with plating medium containing the indicated treatments. After treatments, cells were lysed and luciferase activity was determined using the dual luciferase assay kit (Promega, Madison, WI). Fold change in luciferase activity was calculated after normalization to renilla.

### Anchorage independent growth in soft agar

Anchorage independent growth was determined as described [26]. Briefly, after pouring the base agar (0.6%) layer, the agar was allowed to set. GH3 cells (1.5×10^5^ cells/well, in a 6 well plate) in 2X complete medium (containing either vehicle, ICI 10 nM, or MPP 10 nM) were mixed with 0.3% agar and layered on the bottom gelled layer of agar. Medium (containing ICI/MPP) was changed every 3–4 days, and colony formation was determined by counting the number of colonies using phase-contrast microscopy.

### Western blotting

After treatment cell lysates were harvested as described [20]. Equal amounts of proteins (25–90 µg) were subjected to electrophoresis on 7–12% SDS-PAGE gels. Fractionated proteins were electrophoretically transferred onto PVDF membranes. Incubation of membranes with primary antibodies was done at 4°C o/n. Incubation of membranes with secondary antibodies was done at room temperature for 1 hr, and proteins detected by enhanced chemiluminescence reagents (Pierce), as suggested in the manufacturers protocol.

### Nb2 Bioassay for quantitating PRL release

PRL concentrations in conditioned media (CM) were determined using a bioassay as described [20]. Briefly, after overnight starvation, Nb2 cells were cultured for 72 hr with either rPRL standard, amniotic fluid (1∶1000) serving as an internal control, or with CM aliquots from GH3 cells. After 3 days, Nb2 cell number was determined by the MTT assay as above. The amount of PRL secreted by GH3 cells was calculated from the standard curve and is expressed as µg PRL/µg protein.

### Data analysis

Statistical significance was determined using Student's *t* test, a value of p<0.05 was considered significant.
